# Genetic Association of Curative and Adverse Reactions to Tyrosine Kinase Inhibitors in Chinese advanced Non-Small Cell Lung Cancer patients

**DOI:** 10.1038/srep23368

**Published:** 2016-03-18

**Authors:** Yunfeng Ruan, Jie Jiang, Liang Guo, Yan Li, Hailiang Huang, Lu Shen, Mengqi Luan, Mo Li, Huihui Du, Cheng Ma, Lin He, Xiaoqing Zhang, Shengying Qin

**Affiliations:** 1 Bio-X Institutes, Key Laboratory for the Genetics of Developmental and Neuropsychiatric Disorders (Ministry of Education), Shanghai Jiao Tong University, Shanghai 200030, China.; 2 The Fourth Hospital of Jinan City, Taishan Medical College, Jinan, China.; 3 Centre for Genomic Sciences, the University of Hong Kong, Hong Kong, SAR, China.; 4 Analytic and Translational Genetics Unit, Department of Medicine, Massachusetts General Hospital and Harvard Medical School, Boston, MA 02142, U.S.A.; 5 Broad Institute of Harvard and MIT, Cambridge, MA 02142, U.S.A.; 6 Department of Pharmacy, Shanghai pulmonary Hospital, Tongji University School of Medicine, Shanghai, China.; 7 Shanghai Key Laboratory of Psychotic Disorders, Shanghai Mental Health Center, Shanghai Jiao Tong University School of Medicine, Shanghai, China.

## Abstract

Epidermal growth factor receptor (EGFR) Tyrosine kinase inhibitor (TKI) is an effective targeted therapy for advanced non-small cell lung cancer (NSCLC) but also causes adverse drug reactions (ADRs) e.g., skin rash and diarrhea. SNPs in the EGFR signal pathway, drug metabolism/ transport pathways and miRNA might contribute to the interpersonal difference in ADRs but biomarkers for therapeutic responses and ADRs to TKIs in Chinese population are yet to be fully investigated. We recruited 226 Chinese advanced NSCLC patients who received TKIs erlotinib, gefitinib and icotinib hydrochloride and systematically studied the genetic factors associated with therapeutic responses and ADRs. Rs884225 (T > C) in EGFR 3′ UTR was significantly associated with lower risk of ADRs to erlotinib (*p* value = 0.0010, adjusted *p* value = 0.042). A multivariant interaction four-SNP model (rs884225 in EGFR 3′UTR, rs7787082 in ABCB1 intron, rs38845 in MET intron and rs3803300 in AKT1 5′UTR) was associated with ADRs in general and the more specific drug induced skin injury. The SNPs associated with both therapeutic responses and ADRs indicates they might share a common genetic basis. Our study provided potential biomarkers and clues for further research of biomarkers for therapeutic responses and ADRs in Chinese NSCLC patients.

Non-Small Cell Lung Cancers (NSCLC) make up the major part of lung cancers and are more resistant to chemotherapy and radiation therapy than small cell lung cancers^1^. Previous research has proved that the hyperactivation of epidermal growth factor receptor (EGFR) pathway is the keystone in NSCLC oncogenesis^2^,^3^. EGFR, located on the cell surface, activates proliferative and cell-survival signals by triggering the downstream kinase (such as AKT1)^4^. Based on the above molecular mechanism, targeted drug EGFR tyrosine kinase inhibitors (TKIs) (e.g. erlotinib, gefitinib and icotinib hydrochloride) were developed to treat patients with activating mutations in EGFR^5^ . Clinical trials show that patients with activating mutations in EGFR responded better when treated with TKI than with chemotherapy^6^.

TKIs have a distinguishing adverse drug reaction (ADR) profile from chemotherapy and radiation therapy. They significantly lower the risk of typical severe ADRs to chemotherapy (e.g., neutropenia, thrombocytopenia, anaemia, nausea, constipation, increased ALT, fatigue). However, TKIs increase the risk of skin injury (mainly skin rash) and digestive tract injury (mainly diarrhea)^7^,^8^, both of which still cause considerable discomfort.

Identifying genetic biomarkers for drug response can facilitate personalized medication, which aims to maximize the therapeutic effect and minimize ADRs according to each individual’s profile, e.g., genetic information. So far, studies have mainly focused on the activating mutations in the tyrosine kinase domain of EGFR and have proved that they are predictive biomarkers of therapeutic response to TKIs^9^,^10^,^11^. However the proper biomarkers for TKIs induced ADRs have not yet been fully investigated.

Previous studies have revealed the mechanism of skin rash and diarrhea and their possible correlations with therapeutic responses. The potential for skin rash to be used as a predictor of therapeutic response to TKIs^6^,^12^,^13^ lies in the fact that skin injuries are “on-target” effects caused by the down-stream inhibition of EGFR signaling that interferes the proper function of epidermal cells^14^,^15^,^16^. Unlike skin rash which is the specific response to the inhibition of EGFR signaling, TKI-induced diarrhea is the general result from interference caused by TKI drug molecules^7^.

Evidence has shown that SNPs in the EGFR signal pathway, drug metabolism/ transport pathways and miRNA SNPs might contribute to the interpersonal difference of therapeutic responses and ADRs to TKIs. A gene polymorphism that could influence the EGFR tyrosine kinase signaling might also affect the response to TKIs. Besides the coding SNPs in EGFR, the mutations in the regulation sequences of EGFR (promoter^17^, intron^18^, 5′ UTR^19^) also play a role in carcinogenesis by influencing the expression of EGFR. Moreover, the variations in EGFR 5′UTR have been shown to be associated with skin rash (−216G/T)^19^ and diarrhea (−216 G/T and −191 C/A)^20^ in NSCLC patients.

In addition to the polymorphism of the EGFR gene, mutations in other genes have also been found to influence the EGFR pathway. The activation of hepatocyte growth factor receptor MET mediates resistance to EGFR TKIs^21^. As important regulators of gene expression, miRNAs greatly influence the process of carcinogenesis^22^. Therefore we decided to include miRNA SNPs in our study.

In terms of pharmacokinetics, metabolism (mainly by CYP and UGT family) and transport (mainly by ABC family) of TKIs influenced both therapeutic responses and ADRs. After absorption and distribution, erlotinib and gefitinib are both transported by ATP-binding cassette family protein ABCB1 and ABCG2 and then metabolized in liver by CYP450 family. Erlotinib is metabolized primarily by CYP3A4 and CYP1A1 and marginally by CYP3A5, gefitinib primarily by CYP3A4 and marginally by CYP3A5 and CYP2D6. UGT1A1 is inhibited by erlotinib, CYP2C19 by gefitinib^23^. CYP2C19 has also been reported to be associated with the pharmacokinetics of icotinib hydrochloride^24^.

Studies have found the association between drug metabolism/transport genes and ADRs to TKIs. The polymorphisms of ABCG2 were found to be associated with gefitinib induced diarrhea^25^,^26^. CYP2D6 genotype of reduced activity were associated with gefitinib-induced skin rash^27^. However, a study conducted with 31 Japanese samples found that diarrhea were associated with exposure to gefitinib in plasma but not with common variations in metabolism and transport genes^28^.

So far the pharmacogenetics association studies of TKIs have mainly focused on the single aspect of either therapeutic response or ADRs, and have been conducted mainly among Caucasian populations. In order to facilitate personalized medication among the Chinese population, we conducted biomarker study of therapeutic response and ADRs in 226 Chinese advance NSCLC patients. Based on the previous findings, we selected SNPs from EGFR signal pathway, drug metabolism/ transport pathway and miRNA SNPs for analysis.

## Results

### Patient Characteristics

The general characteristics of the patients are shown in Table 1. The patients who took different TKIs had similar age, progression free survival (PFS), occurrence rate of adverse reaction, objective responses. However, the gender ratio varied in the 3 groups. The patients who had objective response to icotinib hydrochloride showed lower occurrence rate of skin injury but the association between skin rash and therapeutic response still existed among these patients (Table 2).

We found that the therapeutic responses and ADRs were correlated among the patients as shown in Table 2. As expected, PFS and objective response, which are both indicators of therapeutic response, were highly correlated: among the patients who responded, their PFSs were similar no matter which drug they took. The same went with patients who did not respond. Patients who objectively responded to TKIs had approximately 1 year FPS, while PFS of those who did not was approximately 3 months. ADRs, especially skin injury were correlated with therapeutic reactions. However, digestive tract injury was less correlated. This tendency was more obvious among patients who took icotinib hydrochloride.

### SNPs Associated with drug response and adverse drug reactions

**Figure 1:**
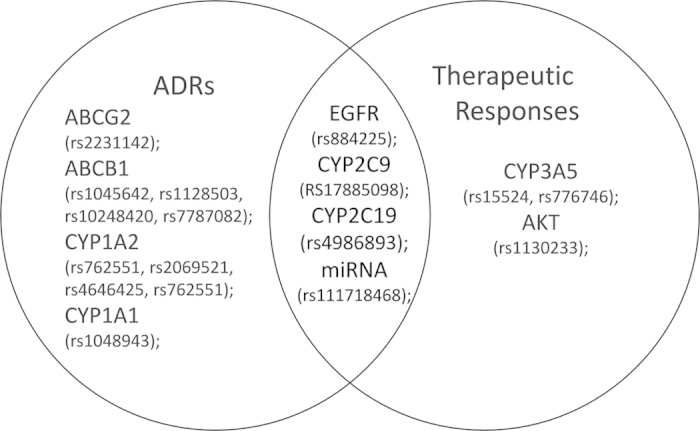
The SNPs associated with therapeutic responses and ADRs.

As shown in Fig. 1, we found 9 SNPs from EGFR pathway and drug metabolism genes associated with objective response, 13 SNPs mainly from drug metabolism and transport genes associated with ADRs. 4 SNPs located in EGFR, CYP2C9, CYP2C19 and miRNA MIR141 were shared by the objective response group and ADR group. However, most associations found in this study did not survive multiple testing correction.

EGFR 3′UTR rs884225 was most significantly associated with both objective response to drug and ADR of all the SNPs analyzed in this study (Table 3). The association of its T > C allele with lower risk of ADR induced by erlotinib survived Bonferroni correction and FDR correction (unadjusted *p* value = 0.0010; adjusted *p* value = 0.042).

For the shared 4 SNPs, the alleles associated with more sensitive objective response were also associated with higher risk of ADR except CYP2C9 rs17885098 (T > C). Rs17885098 T allele was associated with objective response to gefitinib (unadjusted *p* value = 0.049193) while C allele was association with objective response to erlotinib (unadjusted *p* value = 0.0071) and skin injury induced by erlotinib (unadjusted *p* value = 0.0189).

For the 13 SNPs associated with ADRs, only 3 SNPs were associated with digestive tract injury (CYP1A2 SNPs rs2069521 G > A, rs4646425 C > T and miRNA SNP rs111718468).

### Haplotype Associated with adverse drug reactions

After analyzing all the genotyped genes, 3 blocks were identified in ABCB1 (contain rs1045642, rs7787082, rs10248420, 26kb) CYP3A5-CYP3A4 (contain rs15524, rs776746, rs12333983, rs4646440, rs2242480, 115kb) and AKT1 (contain rs2494732, rs1130233, 18kb) respectively. Rs1045642 and rs7787082 in ABCB1 had a strong linkage with D’ = 96, r^2^ = 41; rs15524 and rs776746 in CYP3A5 have a linkage with D’ = 96, r^2^ = 88; rs2494732 and rs1130233 in ATK1 have a linkage with D’ = 94, r^2^ = 42.

As shown in Table 4, only weak association existed between the haplotypes and ADRs. None of the associations was significant after adjustment.

### Multivariant interaction analysis of objective response and adverse drug reaction

We investigated the probable multivariate interactions associated with PFS, objective response, ADRs with multifactor dimensionality reduction (MDR). Of all the possible multivariant models consisting of 2–4 genes, a four-gene model (rs884225 in EGFR 3′UTR, rs7787082 in ABCB1 intron, rs38845 in MET intron and rs3803300 in AKT1 5′UTR) was found to be significantly associated with ADRs as a whole as well as more specific skin injury alone in all the patients undergoing this study (Table 5). None of the 2- and 3-gene models were statistically significant.

## Discussion

TKIs are an effective targeted therapy for advanced NSCLC patients with activating mutations in EGFR but can also cause ADRs, such as skin rash and diarrhea. According to previous findings, the adverse drug reactions (ADRs) of TKIs might be correlated with therapeutic response because of their shared mechanisms. We conducted this study to 1) further identify genetic biomarkers for predicting therapeutic responses and ADRs and 2) analyze the correlation between the therapeutic and adverse responses in Chinese Han population.

In terms of single SNPs analysis, we first identified a strong association between an SNP rs884225 C > T in 3′UTR of EGFR and increased risk of ADR to erlotinib. This association survived Bonferroni correction. SNP rs884225 C > T is very promising potential biomarkers for predicting ADRs to TKIs.

Various studies have shown that activating mutations in the EGFR pathway are associated with improved PFS and improved object response rate. The SNPs in the EGFR promoter and intron were also associated with ADRs to TKIs^19^,^20^, but to our knowledge no association between polymorphism in EGFR 3′ UTR and ADRs to TKIs has previously been found.

A previous study may reveal the mechanism underlying the association between rs884225 and responses to TKIs. Chu *et al*. discovered that rs884225 was significantly associated with bladder cancer risk. According to their bioinformatics analysis, rs884225 polymorphism lay within a predicted binding site for hsa-miR-214, but further *in vitro* validation found that the rs884225(T > C) alone would increase the expression of EGFR, not necessarily by the modulation of hsa-miR-214^29^. We predict that 1) SNP rs884225 might affect the response to erlotinib by influencing the expression of EGFR and 2) this influence might exist in normal tissue cells as well as cancer cells, which would lead to a significant association with ADR and much weaker associations with therapeutic response.

In terms of multiple phenotypes and multigenic analysis, we found that therapeutic responses and ADRs to TKIs are correlated, which accords with previous findings indicating that skin rash could be used as a predictor of therapeutic response to TKIs^6^,^11^,^12^. Digestive tract injuries were less correlated with therapeutic responses.

Although many other SNP associations did not survive multiple testing correction, they could indicate weak associations between SNPs and the phenotypes, which could be further validated with larger sample. First, The SNPs that were associated with both therapeutic and adverse responses indicated that therapeutic and adverse responses might share common genetic basics. Secondly, we assumed that TKIs induced diarrhea might have a genetic basis different from that of skin rash and therapeutic responses. This assumption also accords with our current knowledge that TKIs induced diarrhea might result from general interference caused by TKI molecule^7^ and it is supported by the following evidence: the association between SNPs and digestive injury was weaker than the association between SNPs and skin injury or ADRs as a whole; TKIs induced diarrhea was less correlated with therapeutic responses than TKIs induced skin rash. In addition, previous studies in Caucasian populations found that ABCG2 were associated with diarrhea^25^,^26^ but this finding was not repeated in our study. This indicated that the genetics basic of TKIs induced diarrhea might vary with different populations. From all above, we assume it may be possible to develop other population-specific biomarkers or therapy to reduce the risk of digestive tract injury in the treatment of NSCLC driven by EGFR activating mutations.

We also analyzed multivariant interaction among the EGFR signaling pathways, drug metabolism/transport pathways and miRNA with MDR method. A four-genes model (rs884225 in EGFR 3′UTR, rs7787082 in ABCB1 intron, rs38845 in MET intron and rs3803300 in AKT1 5′UTR) was associated with TKIs induced ADRs and skin rash. The model contains 1 SNP in the drug transport pathway, 2 in the EGFR signaling pathway and 1 in a gene that influences the EGFR pathway. In support for the fidelity of this model, some of the SNPs in this model were associated with other drug responses and oncogenesis. The genotype of rs7787082 in ABCB1 was mildly associated with risk of ADRs to erlotinib in this study (unadjusted *p* value = 0.0356). Allele rs7787082 G was associated with non-response to clozapine in Korean schizophrenia patients^30^. Rs3803300 was associated with risk of schizophrenia and therapeutic response^31^,^32^ and risk of oral squamous cell carcinoma^33^ and survival of early stage NSCLC^34^. This multivariant model indicated that ADRs to TKIs might result from gene interaction among multiple pathways.

In conclusion, we found a strong association between SNP rs884225 and ADR to erlotinib. The multivariant model also indicated that ADRs to TKIs might be regulated by multivariate interactions. These positive results are potential biomarkers for predicting ADRs to TKIs. Other predictions made from our study (e.g. the SNPs that were associated with both therapeutic and adverse responses indicated that therapeutic and adverse responses might share common genetic basis) could serve as guideline for further validation and more in-depth biomarker research. Our study helped to implement personalized medication for Chinse NSCLC patients in terms of both theory and application.

## Subjects and Methods

### Patient recruitment

We recruited 226 NSCLC patients who underwent EGFR TKIs erlotinib, gefitinib and icotinib hydrochloride therapy through our clinical network in Shanghai. We collected their blood sample and clinical records including their gender, age at presentation, cancer family, history, smoking record, cancer diagnosis, pathologic type, stage, medication administration record of adverse reaction and progression free survival (PFS) and blood test results *etc*.

We gained the patients’ informed consent for their participation. The Ethic Committee of Shanghai Ethical Committee of Human Genetic Resources approved this study. Patient recruiting, blood sample collection, clinical information collection and usage were performed according to the guideline and regulation of the committee.

### Genotyping

We genotyped 48 SNP sites in EGFR, AKT1, CMET, CYP1A1, CYP1A2, CYP2C9, CYP2C19, CYP3A4, CYP3A5, UGT1A1, miRNA, ABCB1 and ABCG2. SNP selection were based on the literature review. We predicted the miRNA which possibly influenced the expression of EGFR based on the microRNA database miRBase^35^. Germline genomic DNA was extracted from blood sample with Axygen Blood Genomic DNA Extraction Mini Kit. Genotyping was first performed with MassArray system (Sequenom, CA, USA). The genotyping was designed with Assay Design Suite 2.0 Software. 10–20 ng of genomic DNA was amplified with Gene Amp^®^ PCR system 9700. The PCR product was then processed with iPLEX Gold assay and MassArray System (Sequenom, CA, USA). The SNP sites that were rejected by Assay Design Suite 2.0 were genotyped with ViiA™ 7 System (life Technologies, Carlsbad, California) using TaqMan^®^. The genotyping probes were provided by the Applied Biosystems service. The PCR was performed with TaqMan Universal PCR Master Mix reagent kits in 5ul system (Foster City, California, USA) as the product guideline dictated.

### Data analysis and statistics

The SNPs with success rate <90%, MAF <1% or homogeneous among all the samples were excluded in the following analyses. 40 SNPs were further analyzed (as shown in detail in supplementary file 1).

To reveal the genetic factors that were potentially responsible for different responses to target drugs to NSCLC, we used Response Evaluation Criteria in Solid Tumors (RECIST) system to evaluate the clinical response. We analyzed the association between the patients’ genotypes and objective response to any of the drugs or specific drug (rated “partially response” versus “stable disease” and “progressive disease” in the first month of medication).

For ADRs we divided the patients in case and control group according to their clinical record on adverse drug reactions. The ADRs in our study were either skin injuries (mainly skin rash except one case of paronychia), digestive tract injuries (mainly diarrhea except one case of nausea and one case of nausea and diarrhea), or both.

The discrepancies of allele and genotype frequency of case and control, odds ratios (ORs) and their 95% confidence intervals (CIs), SNP case-control association analysis and Hardy-Weinberg equilibrium were calculated with SHEsis (http://analysis.bio-x.cn/myAnalysis.php). Haplotype block construction was run by Haploview^36^ . The haplotype case-control association study was performed with SHEsis.

Multivariant interaction analyses were performed by multifactor dimensionality reduction (MDR) software^37^. The threshold of statistical significance was p value <0.05 derived from 1000 permutations. The correlation between objective response to TKIs and ADR were calculated with SPSS (http://www-01.ibm.com/software/analytics/spss/).

## Additional Information

**How to cite this article**: Ruan, Y. *et al*. Genetic Association of Curative and Adverse Reactions to Tyrosine Kinase Inhibitors in Chinese advanced Non-Small Cell Lung Cancer patients. *Sci. Rep.* **6**, 23368; doi: 10.1038/srep23368 (2016).

## Supplementary Material

Supplementary Information

## Figures and Tables

**Table 1:**
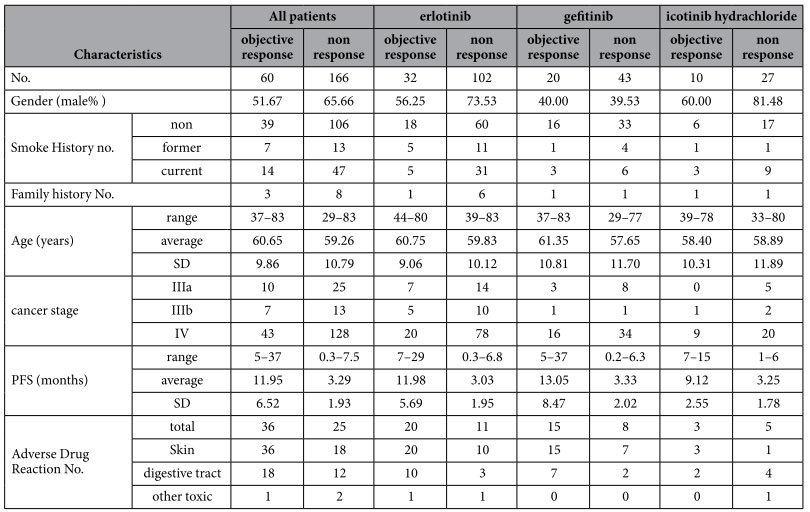
The characteristics of the patients.

SD: standard deviation; No. : the number of.

**Table 2:** The correlation of therapeutic responses and ADRs among patients.

**Drug**		**PFS**	**objective reaction**	**skin injury**
erlotinib	PFS	—	0.766**	—
	ADR	0.559**	0.540**	—
	skin injury	0.559**	0.540**	—
	digestive tract injury	0.533**	0.438**	0.584**
geftinat	PFS	—	0.676**	—
	ADR	0.390**	0.545**	—
	skin injury	0.415**	0.573**	—
	digestive tract injury	0.223	0.404**	0.462**
icotinib hydrachloride	PFS	—	0.801**	—
	ADR	0.120	0.172	—
	skin injury	0.325*	0.376*	—
	digestive tract injury	0.110	0.062	0.555**

^*^p < 0.05,

^**^p < 0.01.

**Table 3:** SNP sites associated with therapeutic responses and ADRs.

Classification	Gene	SNP	P value	phenotype	Genotype number(frequency)			HWE p value
objective response to drug	EGFR	rs884225	0.0226		T T	T C	C C	
				positive	20(0.333)	31(0.517)	9(0.150)	0.5916
				negative	29(0.175)	94(0.566)	43(0.259)	0.0700
	ABCG2	rs2231142	0.0828		A A	A C	C C	
				positive	8(0.138)	32(0.552)	18(0.310)	0.2959
				negative	11(0.067)	79(0.482)	74(0.451)	0.0955
					C C	C T	T T	
objective response to erlotinib	CYP2C9	rs17885098	0.0191	positive	2(0.062)	5(0.156)	25(0.781)	0.0456
				negative	0(0.000)	9(0.088)	93(0.912)	0.6411
					A A	A G	G G	
	CYP2C19	rs4986893	0.0332	positive	1(0.031)	5(0.156)	26(0.812)	0.2628
				negative	0(0.000)	5(0.052)	91(0.948)	0.7934
					A A	A G	G G	
	AKT1	rs1130233	0.0433	positive	2(0.100)	15(0.750)	3(0.150)	0.0243
				negative	14(0.326)	18(0.419)	11(0.256)	0.2981
					C C	C T	C T	
				positive	—	2(0.100)	18(0.900)	0.8139
	miRNA	rs111718468	0.0373	negative	—	0(0.000)	42(1.000)	1.0000
					T T	T C	C C	
ADR to TKIs	EGFR	rs884225	0.0018	positive	23(0.377)	27(0.443)	11(0.180)	0.5367
				negative	26(0.158)	98(0.594)	41(0.248)	0.0111
					C C	C T	T T	
	ABCB1	rs1045642	0.0462	positive	28(0.459)	25(0.410)	8(0.131)	0.5239
				negative	47(0.285)	92(0.558)	26(0.158)	0.0864
					A A	A G	G G	
	ABCB1	rs10248420	0.0434	positive	16(0.271)	30(0.508)	13(0.220)	0.8804
				negative	62(0.403)	76(0.494)	16(0.104)	0.2990
					C C	C T	T T	
ADR to erlotinib	ABCB1	rs1128503	0.0344	positive	4(0.129)	20(0.645)	7(0.226)	0.0922
				negative	12(0.115)	42(0.404)	50(0.481)	0.4889
					T T	T C	C C	
	EGFR	rs884225	**0.0010**	positive	15(0.484)	10(0.323)	6(0.194)	0.1000
				negative	17(0.163)	60(0.577)	27(0.260)	0.0933
					A A	A G	G G	
	ABCB1	rs7787082	0.0356	positive	10(0.323)	11(0.355)	10(0.323)	0.1061
				negative	13(0.125)	51(0.490)	40(0.385)	0.5984
					A A	A C	C C	
	CYP1A2	rs762551	0.0126	positive	7(0.233)	20(0.667)	3(0.100)	0.0503
				negative	50(0.510)	36(0.367)	12(0.122)	0.1805
					A A	A G	G G	
	ABCB1	rs10248420	0.0474	positive	8(0.267)	14(0.467)	8(0.267)	0.7150
				negative	37(0.385)	50(0.521)	9(0.094)	0.1748
ADR to gefitinib	CYP1A1	rs1048943	0.1076		C C	C T	T T	
				positive	3(0.130)	10(0.435)	10(0.435)	0.8416
				negative	1(0.026)	12(0.308)	26(0.667)	0.7804
skin injury induced by TKIs	EGFR	rs884225	0.0073	T T	T C	C C		
				positive	20(0.370)	24(0.444)	10(0.185)	0.5589
				negative	29(0.169)	101(0.587)	42(0.244)	0.0175
					T T	T C	C C	
skin injury induced by erlotinib	EGFR	rs884225	0.0033	positive	14(0.467)	10(0.333)	6(0.200)	0.1221
				negative	18(0.171)	60(0.571)	27(0.257)	0.1211
					C C	C T	T T	
	CYP2C9	rs17885098	0.0222	positive	2(0.067)	4(0.133)	24(0.800)	0.0205
				negative	0(0.000)	10(0.095)	95(0.905)	0.6084
					C C	C T	T T	
	ABCB1	rs1128503	0.0305	positive	3(0.100)	20(0.667)	7(0.233)	0.0503
				negative	13(0.124)	42(0.400)	50(0.476)	0.3750
					A A	A C	C C	
	CYP1A2	rs762551	0.0058	positive	6(0.207)	20(0.690)	3(0.103)	0.0338
				negative	51(0.515)	36(0.364)	12(0.121)	0.1663
					A A	A G	G G	
	CYP2C19	rs4986893	0.0113	positive	1(0.036)	5(0.179)	22(0.786)	0.3311
				negative	0(0.000)	5(0.050)	96(0.950)	0.7987
					A A	A G	G G	
digestive tract injury induced by TKIs	CYP1A2	rs2069521	0.0366	positive	1(0.033)	3(0.100)	26(0.867)	0.0585
				negative	0(0.000)	24(0.123)	171(0.877)	0.3599
					C C	C T	T T	
	CYP1A2	rs4646425	0.0361	positive	26(0.867)	3(0.100)	1(0.033)	0.0585
				negative	172(0.878)	24(0.122)	0(0.000)	0.3613
					C C	C T	C T	
	miRNA	rs111718468	0.0407	positive	—	3(0.100)	27(0.900)	0.7731
				negative	—	5(0.026)	190(0.974)	0.8561

**Table 4:** Haplotypes associated with ADRs.

Phenotype	Haplotype	Case freq. %	Control freq. %	Fisher’s p value	adjusted p value	Odds Ratio [95%CI]
ADRs to TKIs	ABCB1: C A G	41.4	31.5	0.046992	0.23496	1.562 [1.004~2.429]
	ABCB1: T G A	31.4	42.4	0.041685	0.208425	0.626 [0.398~0.984]
Skin injury induced by TKIs	CYP3A5, CYP3A4: C A A C T	4.6	1.2	0.034768	0.17384	3.816 [1.009~14.436]
	AKT: C G	23.8	15.2	0.03813	0.11439	1.755 [1.027~2.999]
ADRs to erlotinib	CYP3A5, CYP3A4: C G T C C	4.8	0.8	0.033093	0.198558	6.511 [0.911~46.559]
Skin injury induced by erlotinib	CYP3A5, CYP3A4: C G T C C	5.0	0.8	0.027835	0.16701	6.835 [0.955~48.917]

**Table 5:** Multivariant interaction of ADRs and skin injury to TKIs.

	P value	CVC	Bal. Acc. CV Training	Bal. Acc. CV Testing	Bal. Acc. Model Training	Bal. Acc. Model Testing	Bal. Acc. Overall
**skin**	0.021	9/10	0.8522	0.6343	0.852	0.6586	0.8472
**ADR**	0.032	9/10	0.835	0.6257	0.8349	0.6676	0.8296

CVC: cross-validation consistency.
