# Supplementary material for: Genetic Association of Curative and Adverse Reactions to Tyrosine Kinase Inhibitors in Chinese advanced Non-Small Cell Lung Cancer patients
Source: Sci Rep. 2016 Mar 18;6:23368. doi: 10.1038/srep23368 (PMC4796893; doi:10.1038/srep23368)
Supplement: Supplementary Information [file srep23368-s1.pdf]

Genetic Association of Curative and Adverse Reactions to Tyrosine Kinase Inhibitors in Chinese advanced Non-Small Cell Lung Cancer patients

Yunfeng Ruan, Jie Jiang, Liang Guo, Yan Li, Hailiang Huang, Lu Shen, Mengqi Luan, Mo Li, Huihui Du, Cheng Ma, Lin He, Xiaoqing Zhang, Shengying Qin

Supplementary file: The SNP sites analyzed in association study and multivariant interaction analysis

Chrome	Position	Gene	rs number
1	40754405	miRNA	rs112439044
2	233760498	UGT1A1	rs4148323
4	88131171	ABCG2	rs2231142
4	88139962	ABCG2	rs2231137
5	55170716	miRNA	rs10061133
7	55147325	EGFR	rs730437
7	55162412	EGFR	rs2293347
7	55206391	EGFR	rs884225
7	87509329	ABCB1	rs1045642
7	87527735	ABCB1	rs7787082
7	87535670	ABCB1	rs10248420
7	87550285	ABCB1	rs1128503
7	87600877	ABCB1	rs3213619
7	99648291	CYP3A5	rs15524
7	99672916	CYP3A5	rs776746
7	99756491	CYP3A4	rs12333983
7	99763247	CYP3A4	rs4646440
7	99763843	CYP3A4	rs2242480
7	99764003	CYP3A4	rs28371759
7	100093806	miRNA	rs72631824
7	116681748	CMET	rs38845
10	94762804	CYP2C19	rs17885098
10	94780653	CYP2C19	rs4986893
10	94781999	CYP2C19	rs72558186
10	94937495	CYP2C9	rs4918758

10	94937702	CYP2C9	rs9332098
10	94981296	CYP2C9	rs1057910
12	6964138	miRNA	rs111718468
14	101041390	miRNA	rs12894467
14	104772855	AKT1	rs2494732
14	104773557	AKT1	rs1130233
14	104803442	AKT1	rs3803300
15	74719300	CYP1A1	rs4646903
15	74720644	CYP1A1	rs1048943
15	74746626	CYP1A2	rs2069521
15	74746892	CYP1A2	rs2069522
15	74749576	CYP1A2	rs762551
15	74750940	CYP1A2	rs4646425
15	74751897	CYP1A2	rs2472304
19	13836478	miRNA	rs895819
